# UAT: Universal Attention Transformer for Video Captioning

**DOI:** 10.3390/s22134817

**Published:** 2022-06-25

**Authors:** Heeju Im, Yong-Suk Choi

**Affiliations:** 1 Department of Artificial Intelligence, Hanyang University, Seoul 04763, Korea; heju0509@hanyang.ac.kr; 2 Department of Computer Science and Engineering, Hanyang University, Seoul 04763, Korea

**Keywords:** video captioning, transformer, end-to-end learning

## Abstract

Video captioning via encoder–decoder structures is a successful sentence generation method. In addition, using various feature extraction networks for extracting multiple features to obtain multiple kinds of visual features in the encoding process is a standard method for improving model performance. Such feature extraction networks are weight-freezing states and are based on convolution neural networks (CNNs). However, these traditional feature extraction methods have some problems. First, when the feature extraction model is used in conjunction with freezing, additional learning of the feature extraction model is not possible by exploiting the backpropagation of the loss obtained from the video captioning training. Specifically, this blocks feature extraction models from learning more about spatial information. Second, the complexity of the model is further increased when multiple CNNs are used. Additionally, the author of Vision Transformers (ViTs) pointed out the inductive bias of CNN called the local receptive field. Therefore, we propose the full transformer structure that uses an end-to-end learning method for video captioning to overcome this problem. As a feature extraction model, we use a vision transformer (ViT) and propose feature extraction gates (FEGs) to enrich the input of the captioning model through that extraction model. Additionally, we design a universal encoder attraction (UEA) that uses all encoder layer outputs and performs self-attention on the outputs. The UEA is used to address the lack of information about the video’s temporal relationship because our method uses only the appearance feature. We will evaluate our model against several recent models on two benchmark datasets and show its competitive performance on MSRVTT/MSVD datasets. We show that the proposed model performed captioning using only a single feature, but in some cases, it was better than the others, which used several features.

## 1. Introduction

Video captioning is one of the notable studies in the computer vision–natural language processing connection. The model understands video and creates captions explaining video via visual data such as frame representation, motion data, and objects. Therefore, the caption represents the information of the video or something changing in the video. Recently, it was revealed that the encoder–decoder architecture is helpful in video captioning. In addition, the architecture, in the previous part of the encoding part, extracts a feature by weight-freezing pre-trained feature extraction models and handles the feature to find the decisive points of the video information. Those methods use not only one kind of feature, such as an appearance feature, but also several kinds of features to deal with more information from videos and process the features in various ways.

Several papers [1,2,3,4] show various methods of captioning. Such video captioning processes typically require a video feature extraction process to convert raw pixel data to the vector form that is required in the entire deep-learning process. Moreover, the pre-trained CNNs have been required for each feature extraction process. For example, in the ORG-TRL [5], the appearance feature that represents frame information is extracted by 2D CNNs, 3D CNNs extract the motion feature, and an object-detection network such as Faster-RCNN extracts the object feature on video.

Because of using pre-trained CNNs to convert a video to features, firstly, the captioning performance is affected by the feature extraction network performance. As can be seen from the experimental results of MGRMP [6], when the network that extracted motion features changed C3D to 3D-ResNext, it showed excellent performance improvement even though it was the same architecture. That proves that good feature extraction significantly influences good capturing performance. Additionally, the E2E Video Captioning [7] proposed the method to optimize the feature extraction network via end-to-end learning.

However, there are some limitations to the traditional feature extraction model. While a weight-freezing network is efficient for feature extraction, it has the disadvantage that it does not update while the entire model is training on new data. In addition, because it is based on CNNs, and since CNNs have a local receptive field, it makes the performance bound. In contrast, the transformer has a global receptive field because of the self-attention layer, improving the model performance when pre-trained well. In many fields [8,9,10], transformer networks outperform CNNs. Therefore, attempting to convert CNN-based feature extraction models to transformer-based models is natural. Inspired by recent studies that apply transformer networks to vision tasks, we propose the full transformer architecture for video captioning. From feature extraction to the part that extracted appearance features, proceeding through to the transformer. We make the model consisting of the (ViT) [11] and adopt end-to-end learning. Moreover, the feature extraction gate (FEG) is proposed to acquire a much better understanding of visual features. The FEG is used to obtain better information and combines CLS token information and previously discarded patch sequence information to extract information that better contains visual content.

Furthermore, we use all encoder layer outputs to resolve the lack of information caused by using one type of feature. As used in the M2 transformer [12], each encoder layer output enters each layer of the decoder as input. In [13], the authors analyzed that each encoder layer output has slightly different information about the relationship between features. Therefore, because each encoder layer output means a different relation of frame feature, we expected the same effect as multi-feature when using multi encoder layer output. At this time, we add additional self-attention to check how the encoder layer outputs are related and to further strengthen the video information. To perform this self-attention, each encoder layer output to be activated must pass through the same network. Thus, the model was designed based on the universal transformer, a layer weight-sharing structure. Furthermore, we named this method the universal encoder layer attention (UEA). In addition, we named our model universal attention transformer (UAT).

Our contributions are the following: (1) we propose the full transformer video captioning structure optimized via end-to-end learning. (2) We design the feature extraction gate (FEG) that considers making better features by a fusion of CLS token and patch sequences. (3) We also propose universal encoder layer attention (UEA), constructed to obtain more information from one feature type.

## 2. Related Work

### 2.1. Video Captioning

Among existing studies, SibNet [4], which uses one type of feature, tried to learn semantic information and content information separately through CNNs, and PickNet [14] proposed a structure that selects and learns frames that are judged to contain important information in a video. MARN [15] proposed an additional memory structure for learning the association between words and video content, SAAT [16] recognized objects and syntax to find actions in the video, and STG-KD [1] identified the movement of objects via spatio-temporal graphs. Additionally, ORG-TRL [5] suggested a way to utilize object features by learning the relationship with surrounding objects through graph convolution.

The latest method, MGRMP [6], used targeting and tracking of important video regions for captioning without using object features. SGN [17] proposed the methods to predict the next-word information through semantic attention within the video. Another latest study, RCG [18], suggested a different method, which was made as a video-text retrieval method, and retrieval was carried out in the generation process.

### 2.2. Vision Transformer

A Vision Transformer [11] proposed a novel method to handle the vision problem via the transformer structure. Many tasks were accomplished with state-of-the-art performance via the ViT. For example, IPT [19] used the transformer on a super-resolution task, and DETR and Swin-TR [9,20] used it on object detection tasks. Additionally, BEiT [21] demonstrated the effectiveness of the transformer on semantic segmentation and outperformed the previous study. These show that the transformer would change all vision tasks’ base architecture.

Furthermore, Arnab et al. [8] proposed a novel architecture to handle video data via a full transformer structure. The authors suggested several model architectures and ”tubelet embedding”, which makes a patch by several frames. In addition, the model structure is two encoder structures, consisting of a spatial and temporal encoder with both enconder layers having the same configuration. Each of the two encoders learns different information. The spatial encoder learns spatial information, and the feature of passing through the spatial encoder is the input of the temporal encoder. Therefore, the temporal encoder analyzes the temporal relation of each spatial feature. As a result, the entire model learns the video’s spatial and temporal information.

### 2.3. Handling Temporal Feature

In the video, not only spatial relationships but also temporal relations are important content because they have rich information about the video, the kinds of motion, and object movement. For example, the authors of [1,22,23] proposed the methods that learn the spatio-temporal relation of video. By that, they obtain state-of-the-art performance in their study. Therefore, learning temporal features, not only spatial features, should be considered to understand the video.

For this, many recent studies, such as OSRG [24] and ORG-TRL [5], and others [25,26] have adopted long short-term memory (LSTM) or recurrent neural networks (RNNs) for the part of their captioning decoder. Traditionally, researchers used these sequence networks to find a sequence’s meaning or to learn about temporal changes in data. Since the temporal feature extraction model, C3D or I3D, is performed to extract the motion features, there is some temporal information. In addition, the appearance features are the set of the video frames, so it is crucial to find the relation between them to obtain information about temporal changes in the video. Therefore, it was natural to adapt these sequence models to the video captioning model.

After the creation of the transformer, researchers tried to use LSTM and RNN but also transformer networks to handle temporal features on video data. In TVT [27], the authors consider the sequence structure of self-attention to frame sequence. By that, the feature of one frame is treated as one temporal feature, and by self-attention, the model learns the overall video by learning the overall temporal relation.

### 2.4. End-to-End Learning

A E2E video captioning [7] proposed an end-to-end learning method on a video captioning structure. The authors show that this encourages encoders to value relevant features for caption generation and their two-stage training strategy. In the first stage, the pre-trained encoder network is frozen, and only the decoder is trained. After several epochs, the entire network is trained end-to-end. First, we consider that strategy. However, because ViViT [8], which the two structure transformer model adapts, is a simple learning method that trains via one stage (no freeze) and shows unblemished performance, we follow that one-stage method.

### 2.5. Universal Transformer

Dehghani et al. [28] proposed the universal transformer structure. These encoders and decoders are weight shared, so there is no more need for additional layer parameters for training. The authors propose the universal structure with a dynamic halting process, but we use only a base universal structure.

For recognizing different layers, time encoding exists on the structure. At each time step, the passing encoder/decoder layers add not only a positional encoding but also a time encoding. By this, the model has just one encoder–decoder layer parameter that could have the effect of learning about different layer features.

## 3. Materials and Methods

**Figure 1:**
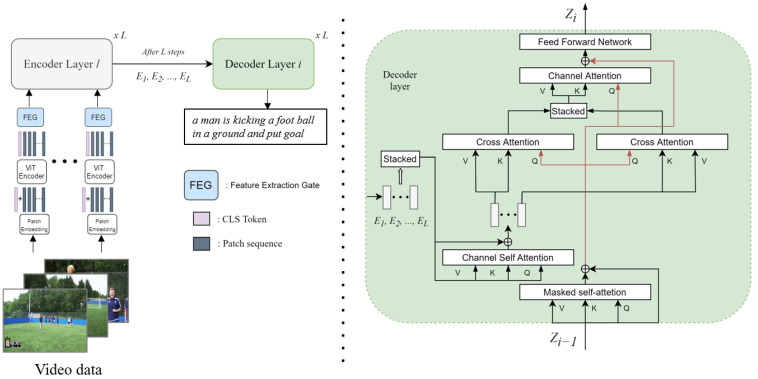
Our full model. The captioning encoder—decoder is the universal transformer structure. Therefore, each encoder and decoder layer is weight-shared. The appearance feature is extracted by ViT, which is our feature extraction model. The decoder layer reads stacked encoder layer outputs after L steps, which means all encoder operations are ended. The left figure describes entire model. The right figure explains our captioning decoder; this includes channel attention and universal encoder attention.

Figure 1 shows the overall architecture. This overall architecture is composed of two models, the feature extraction model and the captioning model. The appearance feature is extracted from the vision transformer. Our approach for the full-captioning model consists of two components. First, it is the feature extraction gate (FEG) that selects a better feature from the ViT. The second is the encoder channel attention on the captioning model. When the model is run, the appearance feature is extracted by the ViT. After that, it passed the FEG and arrives at the captioning encoder. The captioning encoder is in charge of processing and searching for temporal relations from the frame-feature sequence. After that, the captioning decoder reads the output of the encoder layer and generates the captions. In this process, the relationship between video content and the interaction between video content and words are modeled through scaled dot-product attention [29], which exists on the encoder and decoder.

Attention is an operation that performs a weighted sum with a value vector by scoring the similarity of the query and key distribution. Since our model consists of a full transformer structure, attention is performed everywhere. The scaled-dot product attention operation can be defined as follows:

(1) $$\mathrm{Attention}\left(Q,K,V\right)=\mathrm{softmax}\left(\frac{QK^{T}}{\sqrt{d}}\right)V$$

where *Q* is a matrix consisting of $n_{q}$ query vectors and *K* and *V*; both the matrices consist of $n_{k}$ keys and values. *Q*, *K*, and *V* all have the same dimension, and *d* is a scaling factor.

Additionally, there is multi-head attention (MHA), which calculates the new expression of *h* times in the context of the whole context. The idea of MHA is acquiring *h* new expressions that reflect the context and using the matrix by concatenating these various expressions as the attention output. It is formulated as:

(2) $$\mathrm{MHA}\left(F,F,F\right)=\mathrm{concatenate}\left({\mathrm{head}}_{1},\ldots ,{\mathrm{head}}_{h}\right)W_{0}$$

(3) $${\mathrm{head}}_{i}=\mathrm{Attention}\left(FW_{i}^{Q},FW_{i}^{K},FW_{i}^{V}\right)$$

(4) F=LayerNormalization(I)

where $W_{i}$ is a trainable matrix and *h* is the number of heads. Our layer normalization is performed before MHA operates. Therefore, the inputs of MHA and *F* are the normalized *I*, which are input features.

### 3.1. Feature Extraction Model

**ViT Feature Extraction Process.** Firstly, we extract *T* frames from the video. Then, each frame is passed into the transformer encoder for feature extraction. At this time, in order to input the given input pixel data to sequence data, which is an input of the ViT, it must be reshaped by patch embedding. Each pixel datum is divided into a fixed patch size *P*, and the frame features are reshaped to form a sequence where $N=\frac{HW}{P^{2}}$. Furthermore, the made patch has the dimensions $R^{P^{2}\times C}$. By embedding, it has $d_{model}$ dimension size and becomes $X_{0}\in R^{N\times d_{model}}$.

After performing concatenation on the one token, a learnable positional embedding is added and the transformer encoder is entered. This token is called the CLS token. The encoder layer mechanism is defined as:

(5) $$\begin{array}{l} \dot{X_{k}}=\mathrm{MHA}\left(\left(X_{k-1}\right),\left(X_{k-1}\right),\left(X_{k-1}\right)\right)+X_{k-1},\\ X_{k}=\mathrm{FFN}\left(\mathrm{LN}\left(\dot{X_{k}}\right)\right)+\dot{X_{k}},\\ \mathrm{FFN}\left(x\right)=\mathrm{RELU}\left(xW_{1}+B_{1}\right)W_{2}+B_{2} \end{array},$$

where k=0,…,M; *M* is the number of feature extraction model layers. LN means layer normalization. FFN is a feed-forward network that consists of a ReLU function and a fully connected layer. The output calculated in this way has N x D, which is equal to the shape of the input.

**Figure 2:**
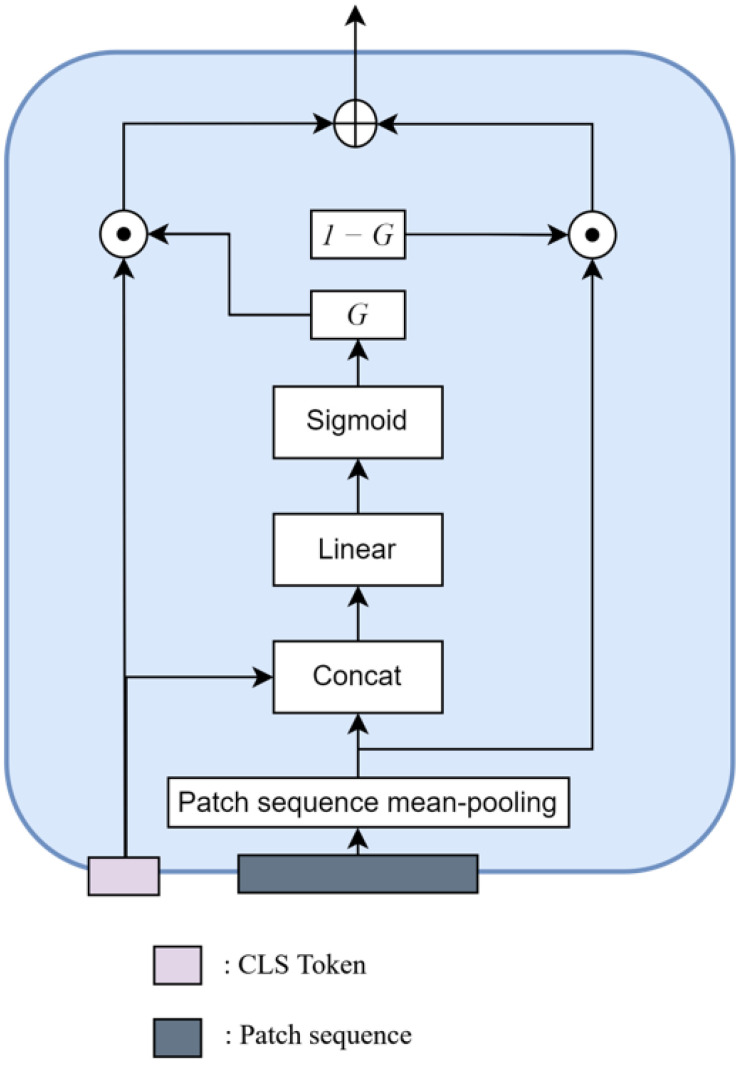
The architecture of the feature extraction gate (FEG). This generates the captioning model input by controlling the ratio of the CLS token feature and the mean-pooled patch sequence feature.

**Feature Extraction Gate.** As shown in Figure 2, unlike other existing methods that use only CLS tokens, we consider using the entire output sequence to make better features. First, we perform avg-pooling on the patch sequence $R^{N\times D}$ to make the same shape as the CLS token. After that, the weighted sum is performed, and the feature passes through the sigmoid function to create the gate feature ‘*G*’, which has a value between 0 and 1. This *G* determines which information to take from the CLS token. Likewise, 1−G is used to control the avg-pooled feature sequence. After that, we add two features after an element-wise multiplication of *G* with the CLS token and 1−G with the pooled frame sequence. In this way, features with the shape of $R^{1\times D}$ are obtained that combine not only CLS token information but also patch features information in one frame. Namely, this gate structure compares features and makes the fusion of the CLS token and the patch sequence. It is formulated as:

(6) $$\dot{G}=\mathrm{concatenate}\left(X_{CLS},X_{pooled}\right)W_{3}$$

where $W_{3}\in R^{{2d}_{model}\times d_{model}}$ represents the trainable weights. The two features $X_{CLS},X_{pooled}$ are concatenated and sum-weighted. After making the gate feature, it passes the sigmoid function. That $G_{sig}$ is calculated with $X_{CLS}$, and the 1−G is calculated with $X_{pooled}$. The formula is as follows:

(7) $$G=\mathrm{sigmoid}(\dot{G})$$

(8) $$F=X_{CLS}⊙G+\left(1-G\right)⊙X_{pooled},$$

where ⊙ means an element-wise multiplication. We named this module the feature extraction gate (FEG).

### 3.2. Captioning Model

**Captioning Encoder.** Our captioning encoder has a role in analyzing the temporal relation of the extracted frame features which ViT makes. The input sequence length in the captioning part is T, the same as the number of keyframes. In this process, the relationship of keyframes that appears in temporal information of video content is learned from the captioning encoder. Because the captioning encoder is the same structure as the feature extraction encoder, the encoder performs the attention operation, similar to the feature extraction encoder. However, we need additional positional embedding to learn temporal features. Since our feature extraction model is the ViT that embeds patches about a 1-frame image and performs the attention operation to spatial information, it only performs spatial embedding. Therefore, we add a positional embedding to the output of FEG to make the model learn temporal relationships.

Next, we stacked all encoder outputs. These stacked encoder layer outputs are as follows:

(9) $$E_{s}=\mathrm{stacked}\left(E_{l}\right)$$

where l=1,…,L; *L* is the number of layers. This stacked feature is used in the captioning decoder.

**Captioning Decoder.** $Z_{p_{i}}$ is the result of the masked multi-head self-attention of $Z_{i-1}$. $Z_{0}$ is the embedded vector of the target words, where $Z_{0}\in R^{W\times d_{model}}$. *W* is the maximum length of a sentence. Next, the second MHA on the decoder generates the channel attentive feature. The formula is:

(10) $$\begin{array}{c} Z_{p_{i}}=\mathrm{MHA}\left(Z_{i-1},Z_{i-1},Z_{i-1}\right)+Z_{i-1} \end{array}$$

(11) $$\begin{array}{c} E_{CA^{i}}=\mathrm{MHA}\left(E_{s},E_{s},E_{s}\right)+E_{s} \end{array}$$

where i=1,…,L; $E_{CA^{i}},E_{s}\in R^{T\times L\times d_{model}}$. We named the MHA operation that performs for $E_{s}$, Equation (10), as the channel self-attention (CSA). We construct this CSA with the residual connections, so after MHA is performed, the query vector is summed to the output vector. Additionally, the attention layer input is normalized by layer normalization before the attention operates, as mentioned in Equation (4).

Equation (12) is performed to make the attentive features of $Z_{p_{i}}$ for each channel attentive feature $E_{CA_{l}^{i}}$. When cross multi-head attention is running, $E_{CA_{l}^{i}}$ is calculated with $Z_{p_{i}}$ and made into a new vector. This time, the query vector $Z_{p_{i}}$ is not summed. After it is finished, each $E_{CA_{l}}^{i}$ is made and stacked once more, and $E_{all}^{i}$ is made. In addition, because the values are accumulated as many times as there are encoder layers, multi-head channel attention is performed to obtain the attention value with the same size of the query vector $Z_{p_{i}}$. On the multi-head channel attention, if each encoder layer is considered one channel, each channel’s attention score is calculated. The result $Z_{o_{i}}$ reflects each channel as much as the corresponding score is obtained. Finally, the decoder output $Z_{i}$ is obtained by layer normalizing and passes through the FFN. By this, the entire cross attention is performed. The entire mechanisms are defined as:

(12) $$\begin{array}{c} E_{CA_{l}}^{i}=\mathrm{MHA}\left(Z_{p_{i}},E_{CA_{l}^{i}},E_{CA_{l}^{i}}\right) \end{array}$$

(13) $$\begin{array}{c} E_{all}^{i}=\mathrm{stacked}\left(E_{CA_{l}}^{i}\right) \end{array}$$

(14) $$\begin{array}{c} Z_{o_{i}}=\mathrm{MHA}\left(Z_{p_{i}},E_{all}^{i},E_{all}^{i}\right)+Z_{p_{i}} \end{array}$$

(15) $$\begin{array}{c} Z_{i}=\mathrm{FFN}\left(\mathrm{LN}\left(Z_{o_{i}}\right)\right)+Z_{o_{i}} \end{array}$$

### 3.3. Universal Structure

We propose a encoder layer attention by channel self-attention. However, the problem is that each encoder is independent, so each encoder layer outputs come from a different layer. The self-attention mechanism creates a new value by comparing and scoring how many results from the same model are related. Therefore, it is pointless to perform self-attention with layers from each different encoder layer. To overcome this problem, we adopt the universal transformer structure.

The universal transformer is a weight-shared structure. Each encoder and decoder layer parameter is weight-shared, meaning the encoder layers’ outputs pass to the next encoder layer, which has the same parameters. After the L step, where L is the number of layers, the encoder outputs are stacked and proceed toward the decoder. The universal structure is defined as:

(16) $$\begin{array}{c} E_{L}^{i}={\mathrm{Universal}\mathrm{Encoder}\mathrm{Layer}}^{i}\left(E_{L}^{i-1}\right),\\ E_{stacked}=\mathrm{stacked}\left(E_{L}^{i}\right),\\ Z_{l}={\mathrm{Universal}\mathrm{Decoder}\mathrm{Layer}}^{l}\left(E_{stacked},Z_{l-1}\right), \end{array}$$

where $E_{L}^{i}\in R^{T\times d_{model}}$ and $E_{L}^{0}$ is the first input of the universal encoder, *F*. Because all output comes from the same layer, channel self-attention performs well, so it could be helpful to find useful features. Moreover, we construct the decoder on a universal network for parameter balance with the encoder.

We defined this method as the universal encoder attention (UEA) that applies CSA, as mentioned in the captioning decoder section, to the universal encoder layer outputs.

## 4. Results

### 4.1. DataSet

The Microsoft Video Description Corpus (MSVD) [30] is a widely used video captioning benchmark dataset. It is composed of 1970 videos and multilingual sentences. On average, each video has 40 English sentences, and we use them all. Following prior work [31], we split the dataset to 1200/100/670. A total of 1200 videos were used for training, and 100 sets were used for validation. The remaining videos were used for testing.

Microsoft Research Video to Text (MSR-VTT) [32] comprises 10,000 video clips from 20 categories, such as sports, movies, and music. Each clip is annotated with 20 English captions made by Amazon Mechanical Turks. Previous works split the dataset into 6513 clips for training and 497 clips for validation, and the others were used for testing. We followed that division. The average sentence length is 20 words.

### 4.2. Metrics

To evaluate, we used four metrics, BlEU-4 [33], METEOR [34], ROUGE-L [35], and CIDEr [36]. The BLEU-4 metric scores the precision of four grams between ground truth and prediction. METEOR measures the F-score, a penalty function for incorrect words. Another metric, ROUGE-L, uses the longest common subsequence (LCS) for scoring. The CIDEr score is obtained by computing cosine similarity to all ground truth sentences and averaging its score.

### 4.3. Implementation Details

We uniformly sampled eight keyframes from all videos. All keyframes were resized to 224 × 224. The full model was trained over eight epochs in one stage via end-to-end learning, when the captioning model was a universal structure. If the vanilla transformer structure was adopted, we trained 15 epochs. In addition, the model was learned to minimize cross-entropy loss. We used the Adam optimizer [37] with a fixed learning rate of $2\times 10^{-5}$, and the beam search with a beam size of five. The batch size was eight.

We employed the pre-trained ViT-Base model, which was pre-trained on ImageNet-21k, as the feature extraction model. The patch size was 16, and other details follow on ViT [11]. Our captioning model layers were four, and the attention head was eight. A 0.3 dropout ratio was used. $d_{model}=768$ was the embedding dimension size, which was the same as the hidden dimension size. We selected the test model from the best performance on the validation.

### 4.4. Performance Comparison

To evaluate our method, we compare it with the previous methods. Table 1 shows the quantitative results on MSR-VTT and MSVD. Additionally, to compare our approach to using the ViT and only appearance features with other methods, we list the appearance, motion, and object features with extraction models. Reinforcement learning is not used for a fair comparison.

In both the MSVD and MSR-VTT datasets, our model obtained a significant improvement on the BLEU-4 score. Specifically, this is a very encouraging performance, considering that only the appearance feature was used. Moreover, other single-feature models’ results, such as those of PickNet [14], TVT [27], and RecNet [38], on the MSVD and MSR-VTT datasets have shown that the CIDEr score is relatively low compared to the BLEU-4 score. Our modelm however, achieves a higher BLEU-4 score and CIDEr score simultaneously. In addition, VRE [39]’s MSRVTT dataset experimental results using audio information together showed better performance on several metrics than our model, but our model performed much better in MSVD, an environment without audio information. Furthermore, it obtained a higher CIDEr score than the methods using multi-features such as MARN [15], OA-BTG [40], POS+VCT [41], and SAAT [16]. Unlike the others, OSRG [24] adopted MaskTrack-RCNN [42] to extract the bounding box of the object and obtain motion information of objects. Additionally, the authors used adversarial reinforcement learning (ARL) to train. By the method, OSRG achieves state-of-the-art performance on four metrics. Although this method shows very high performance, we do not directly compare it with our model because it used reinforcement learning and tracking models in the feature extraction process. In addition, Ref. [43] proposed a new method and achieved state-of-the-art performance for the best sentences in the video captioning process. However, unlike other methods, this method uses multiple sentence generation techniques and evaluates model performance, so we did not make performance comparisons with that model.

Additionally, we compare our models to TVT [27], on which the captioning model is constructed, to a vanilla transformer and the features extracted by NasNet [44] and I3D [45]. Our base model performance is shown in Table 2. TVT (Base) is similar to our base model, except for the end-to-end learning and the feature extraction model. It has a higher score than ours on BLEU-4, but shows less performance on others. TVT (Att) uses motion features through methods such as channel attention. They pass appearance/motion features between different encoders and perform cross attention separately, then stacking and fusing them, as with our channel attention. Although multi-features are used for the captioning structure of the same vanilla transformer, TVT does not significantly outperform our base model.

**Table 1:** Comparison performance on MSRVTT/MSVD. Features show the feature extraction models on each method. B@4, M, R, and C mean the BLEU-4, METEOR, ROUGE-L, and CIDEr metrics. We assigned the feature extraction network that each model used. IRv2 is InceptionResnetV2 and MT-RCNN is Masktrack-RCNN.

Method	Features			MSRVTT				MSVD			
	Appearance	Motion	Object	B@4	M	R	C	B@4	M	R	C
PickNet [14]	ResNet-152	-	-	39.4	27.3	59.7	42.3	52.3	33.3	69.6	76.5
RecNet [38]	GoogleNet	-	-	39.1	26.6	59.3	42.7	52.3	34.1	69.8	80.3
SibNet [4]	GoogleNet	-	-	40.9	27.5	60.2	47.5	54.2	34.8	71.7	88.2
TVT(Base) [27]	NasNet	-	-	38.0	27.1	58.8	45.6	52.5	34.4	70.1	75.9
TVT(Att) [27]	NasNet	I3D	-	40.1	27.9	59.6	47.7	53.0	34.7	71.7	80.8
OA-BTG [40]	ResNet-200	-	MaskRCNN	41.4	28.2	-	46.9	56.9	36.2	-	90.6
MARN [15]	ResNet-101	3D-ResNext-101	-	40.4	28.1	60.7	47.1	48.6	35.1	71.9	92.2
VRE [39]	ResNet-152	-	-	43.2	28.0	62.0	48.3	51.7	34.3	71.9	86.7
POS-VCT [41]	IRv2	C3D	-	42.3	29.7	62.8	49.1	52.8	36.1	71.8	87.8
SAAT [16]	IRv2	C3D	FasterRCNN	39.9	27.7	61.2	51	46.5	33.5	69.4	81
STG-KD [1]	ResNet-101	3D-ResNext-101	FasterRCNN	40.5	28.3	60.9	47.1	52.2	36.9	73.9	93
ORG-TRL [5]	IRv2	C3D	FasterRCNN	43.6	28.8	62.1	50.9	54.3	36.4	73.9	95.2
RCG [18]	IRv2	C3D	-	42.8	29.3	61.7	52.9	-	-	-	-
SGN [17]	ResNet-101	3D-ResNext-101	-	40.8	28.3	60.8	49.5	52.8	35.5	72.9	94.3
MGRMP [6]	IRv2	3D-ResNext-101	-	41.7	28.9	62.1	51.4	55.8	36.9	74.5	98.5
TTA [46]	ResNet-152	C3D	-	41.4	27.7	61.1	46.7	51.8	35.5	72.4	87.7
OSRG [24]	IRv2	MT-RCNN	MT-RCNN	46.5	33.6	65.6	54.3	59.8	38.5	88.2	97.8
Ours	ViT-B/16	-	-	43.0	27.8	60.9	49.7	56.5	36.4	72.8	92.8

Moreover, our method used few keyframes, relatively. STG-KD [1] uses 10 keyframes, and RCG [18], SAAT [16], OSRG [24], and ORG-TRL [5] use 28 keyframes. In addition, MGRMP [6] uses 32 keyframes for extracting the appearance feature. On the other hand, our model uses eight keyframes. Despite the use of these few keyframes, our model achieves better performance than some of the above models.

### 4.5. Ablation Studies

**Role of FEG and UEA.** We compare our model separately. The experiment results are shown in Table 2. (1) Base model is the model which consists of two components: ViT and the vanilla transformer. (2) w FEG, w/o UEA is the model adding the FEG to the base model. (3) w/o FEG, w UEA is the model without FEG, and which adds UEA. (4) w FEG, w UEA is our full model, with FEG and UEA to check the effects on each module. It is shown that the performance improvement is insufficient when the FEG is attached to the vanilla transformer. However, the model with the FEG definitely obtains higher scores than the model without the FEG when the FEG is used with the UEA. This is demonstrated by comparing (3) and (4). That means the FEG is more effective when used with the UEA.

**Universal approaches.** Additionally, we test the universal captioning model to check if the effect of UEA is better than other methods. All the models have experimented without the FEG. Univ-Base is the vanilla universal transformer. Univ-EA means our UEA model. Table 3 shows the results. The Univ-Base model achieves a high BlEU-4 score. However, it has a low score on the CIDEr metric, and Univ-Full shows an improvement on the CIDEr metric over the Univ-Base model. This result means the universal structure makes a model obtain a high BlEU-4 score, and UEA complements the lack of a CIDEr score.

**Effect of the number of layers.** We explore UEA performance when the number of encoder layers is different. Table 4 shows the results with two, three, four, five, and six layers. Note the results when two and four layers are used. This demonstrates that our approach is fine to generate precise captions. On the other hand, when six layers are used, the performance is lower than that of using four layers. This shows that when the layers were too much, some encoder layer outputs could not be found regarding the meaningful video sequence relationship, and disturbed the model to learn from the other significant outputs.

When the number of layers is five, it shows the best BLEU-4 score and CIDEr score. However, because it has a low ROUGE-L score, we chose the four-layer structure in other experiments. Therefore, all models tested in the ablation study are constructed into four layers.

**Freezing the feature extraction model.** Moreover, we tested our model with a weight-frozen ViT to reveal the effect of end-to-end learning. Therefore, all features were extracted by the ViT pre-trained on ImageNet-21k. On captioning parts, the base model is composed of a vanilla transformer, but our model consists of the FEG and the UEA. Table 5 shows the results on the MSVD dataset.

Comparing Table 2 and Table 5, the base model shows a higher CIDEr score when using weight-frozen ViT compared to end-to-end learning. It shows end-to-end learning does not improve the captioning performance greatly when using a vanilla transformer. On the other side, our model performance is higher on all metrics than they are when using weight-freezed ViT. The results mean our model is more suited for the end-to-end learning method than the vanilla transformer.

**Change Feature Extraction Model.** We test our model with CNN feature extraction networks. We adopt ResNet152 [47] for the CNN feature extraction model and our UEA module for the captioning model. In Table 6, we compare this model with a vanilla transformer. Even though the appearance feature is only used and the motion feature is not, UEA makes the model perform better than the Base. This shows that using UEA also has a positive effect on CNN feature extraction-based video captioning.

### 4.6. Qualitative Analysis

**Figure 3:**
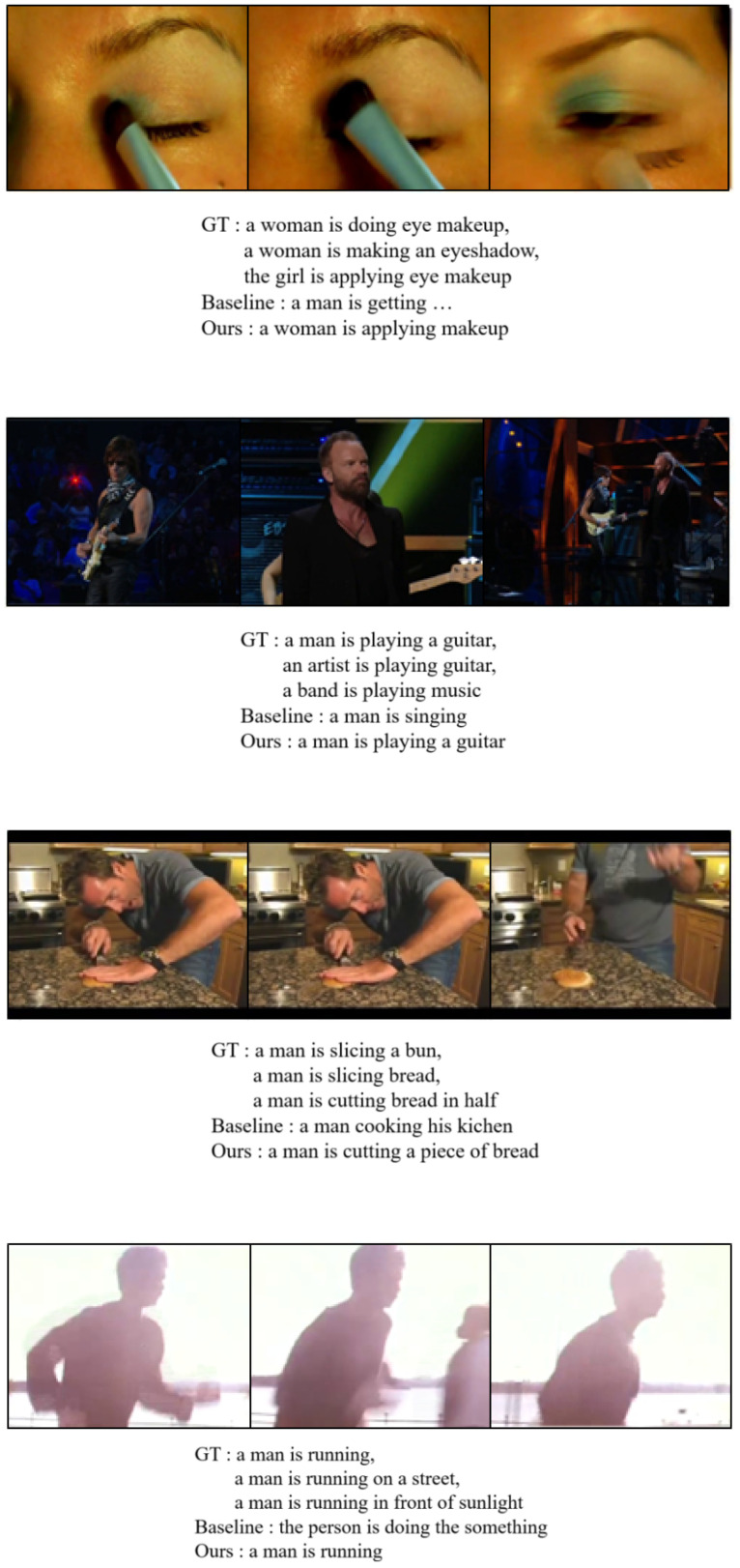
Qualitative results of our model and the base model on the MSVD dataset. Each case shows that our model is better than the base model. This shows that FEG and UEA operate well to generate an actual sentence.

**Figure 4:**
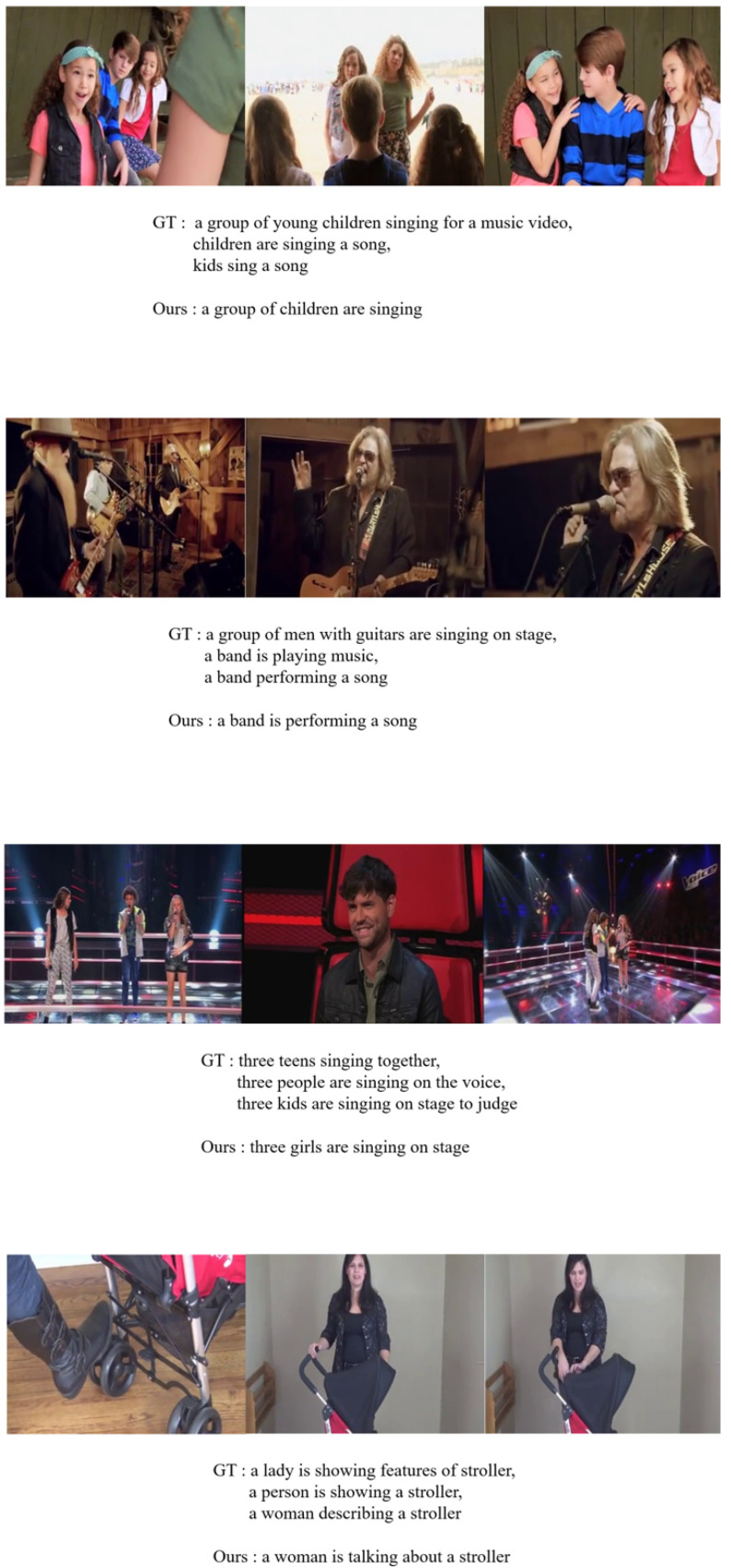
Qualitative results on the MSRVTT dataset.

We show qualitative results with our model and the baseline model. This baseline model means the ViT + vanilla transformer without the FEG and the UEA. “Ours” means our full model with the FEG and the UEA. Figure 3 shows that the baseline model could not catch some important words. However, our model catches words such as “running”, “makeup”, and “bread”. This shows that our approach affects the model performance directly. Figure 4 shows qualitative results on the MSRVTT dataset.

## 5. Discussion

In this paper, we have proposed a novel video captioning structure consisting of a complete transformer with a novel attention method. Particularly, we changed the backbone CNNs to transformer networks and checked the effect of the model via end-to-end learning. The feature extraction gate and additional attention methods make the feature more effective in improving performance. In addition, our model uses only the appearance feature which is extracted from the image frame. It is encouraging that our model, which is trained by using only frame information, shows better performance than the SAAT and STG-KD [1,16] models, which achieve performance scores by using several kinds of visual features, such as motion and object features extracted from other CNNs. We will explore the novel method to use multiple kinds of visual features, such as object features, via a new transformer architecture. 

## Figures and Tables

**Table 2:** Ablation study with the FEG. On the base model, performance improvement by the FEG is insignificant.

Method	B@4	M	R	C
Base model	47.8	35.3	71.5	81.5
w FEG	47.5	35.5	70.7	82.5
w UEA	52.6	34.9	71.2	84.1
w FEG, w UEA	56.5	36.4	72.8	92.8

**Table 3:** Ablation studies for universal structure.

Method	B@4	M	R	C
Univ-Base	55.0	35.2	71.6	80.8
Univ-EA	52.6	34.9	71.2	84.1

**Table 4:** Results on the number of layers.

Layers	B@4	M	R	C
Two layers	55.0	35.2	71.6	85.5
Three layers	55.2	36.2	72.8	90.6
Four layers	56.5	36.4	72.8	92.8
Five layers	56.9	36.4	71.7	93.8
Six layers	54.1	36.0	71.8	87.7

**Table 5:** Results on the base model and our model. Those two experiment are performed on ViT with weight freezing.

Method	B@4	M	R	C
Base	47.7	35.1	70.4	86.6
Ours	54.9	36.4	72.7	92.5

**Table 6:** Ablation studies for UEA. We compare the performance effect of UEA with the CNN feature extraction model (ResNet152). On experiments, we froze the feature extraction model. The captioning model was tested on the MSVD dataset. Base means vanilla transformer model.

Model	B@4	M	R	C
ResNet152 + Base	46.53	31.75	66.72	77.30
ResNet152 + UEA	48.34	33.40	68.89	82.49

## Data Availability

The MSVD dataset can be downloaded from https://www.cs.utexas.edu/users/ml/clamp/videoDescription/. The MSRVTT dataset can be downloaded from https://www.mediafire.com/folder/h14iarbs62e7p/shared.
